# MASH Native: a unified solution for native top-down proteomics data processing

**DOI:** 10.1093/bioinformatics/btad359

**Published:** 2023-06-09

**Authors:** Eli J Larson, Melissa R Pergande, Michelle E Moss, Kalina J Rossler, R Kent Wenger, Boris Krichel, Harini Josyer, Jake A Melby, David S Roberts, Kyndalanne Pike, Zhuoxin Shi, Hsin-Ju Chan, Bridget Knight, Holden T Rogers, Kyle A Brown, Irene M Ong, Kyowon Jeong, Michael T Marty, Sean J McIlwain, Ying Ge

**Affiliations:** Department of Chemistry, University of Wisconsin–Madison, Madison, WI 53705, United States; Department of Cell and Regenerative Biology, University of Wisconsin–Madison, Madison, WI 53705, United States; Department of Cell and Regenerative Biology, University of Wisconsin–Madison, Madison, WI 53705, United States; Department of Cell and Regenerative Biology, University of Wisconsin–Madison, Madison, WI 53705, United States; Department of Cell and Regenerative Biology, University of Wisconsin–Madison, Madison, WI 53705, United States; Human Proteomics Program, School of Medicine and Public Health, University of Wisconsin–Madison, Madison, WI 53705, United States; Department of Cell and Regenerative Biology, University of Wisconsin–Madison, Madison, WI 53705, United States; Department of Cell and Regenerative Biology, University of Wisconsin–Madison, Madison, WI 53705, United States; Department of Chemistry, University of Wisconsin–Madison, Madison, WI 53705, United States; Department of Chemistry, University of Wisconsin–Madison, Madison, WI 53705, United States; Department of Chemistry, University of Wisconsin–Madison, Madison, WI 53705, United States; Department of Cell and Regenerative Biology, University of Wisconsin–Madison, Madison, WI 53705, United States; Department of Chemistry, University of Wisconsin–Madison, Madison, WI 53705, United States; Department of Chemistry, University of Wisconsin–Madison, Madison, WI 53705, United States; Department of Chemistry, University of Wisconsin–Madison, Madison, WI 53705, United States; Department of Chemistry, University of Wisconsin–Madison, Madison, WI 53705, United States; Department of Biostatistics and Medical Informatics, University of Wisconsin–Madison, Madison, WI 53705, United States; University of Wisconsin Carbone Cancer Center, University of Wisconsin-Madison, Madison, WI 53705, United States; Department of Obstetrics and Gynecology, University of Wisconsin–Madison, Madison, WI 53705, United States; Department of Applied Bioinformatics, University of Tübingen, Tübingen 72704, Germany; Department of Chemistry and Biochemistry, University of Arizona, Tucson, AZ 85719, United States; Department of Biostatistics and Medical Informatics, University of Wisconsin–Madison, Madison, WI 53705, United States; University of Wisconsin Carbone Cancer Center, University of Wisconsin-Madison, Madison, WI 53705, United States; Department of Chemistry, University of Wisconsin–Madison, Madison, WI 53705, United States; Department of Cell and Regenerative Biology, University of Wisconsin–Madison, Madison, WI 53705, United States; Human Proteomics Program, School of Medicine and Public Health, University of Wisconsin–Madison, Madison, WI 53705, United States

## Abstract

**Motivation:**

Native top-down proteomics (nTDP) integrates native mass spectrometry (nMS) with top-down proteomics (TDP) to provide comprehensive analysis of protein complexes together with proteoform identification and characterization. Despite significant advances in nMS and TDP software developments, a unified and user-friendly software package for analysis of nTDP data remains lacking.

**Results:**

We have developed MASH Native to provide a unified solution for nTDP to process complex datasets with database searching capabilities in a user-friendly interface. MASH Native supports various data formats and incorporates multiple options for deconvolution, database searching, and spectral summing to provide a “one-stop shop” for characterizing both native protein complexes and proteoforms.

**Availability and implementation:**

The MASH Native app, video tutorials, written tutorials, and additional documentation are freely available for download at https://labs.wisc.edu/gelab/MASH_Explorer/MASHSoftware.php. All data files shown in user tutorials are included with the MASH Native software in the download .zip file.

## 1 Introduction

Native mass spectrometry (nMS) analyzes intact proteins and protein complexes under non-denaturing conditions to preserve their tertiary structure and non-covalent interactions in the gas phase, which has emerged as a powerful structural biology tool to define protein structure–function relationships (Loo 1997, Sharon and Robinson 2007, Leney and Heck 2017, Keener *et al.* 2021, Karch *et al.* 2022). Native top-down proteomics (nTDP) integrates nMS with top-down proteomics (TDP) (Catherman *et al.* 2014; Toby *et al.* 2016; Chen *et al.* 2018; Melby *et al.* 2021), which enables structural characterization of protein complexes together with proteoform sequencing to locate non-covalent ligand binding sites, posttranslational modifications, and mutations (Li *et al.* 2018; Zhou *et al.* 2020; Karch *et al.* 2022; Jooß *et al.* 2022). nTDP first measures intact proteins and protein complexes under non-denaturing conditions (MS1) then directly fragments proteins and protein complexes in the gas phase (MS2) to obtain primary sequence information from a single dissociation event (Li *et al.* 2018). Alternatively, nTDP may be implemented in the “complex-down” mode using two separate dissociation events: (1) dissociation of intact protein complexes (MS1) into protein subunits (MS2') by low-energy collision-induced dissociation (CID) or surface-induced dissociation, and (2) fragmentation of subunits (MS3) by tandem mass spectrometry techniques such as high-energy CID, electron capture dissociation, electron transfer dissociation, or ultraviolet photodissociation to provide primary sequence coverage and localize modifications (Skinner *et al.* 2018b; Stiving *et al.* 2019; Jooß *et al.* 2022).

Currently one of the major challenges in nTDP is the analysis of complex nTDP datasets which include both isotopically resolved and isotopically unresolved MS1 and MS2' spectra as well as the complicated MS2 and MS3 data, and difficulties in database searching. Although multiple software packages have been developed for nMS of known proteins and complexes (Marty *et al.* 2015; Cleary *et al.* 2016, 2018; Reid *et al.* 2019), the lack of any MS2/MS3 fragmentation assignment and database searching prevent the identification of unknown proteins. Meanwhile, significant efforts have been allocated toward the development of software packages for denatured TDP with capability in analyzing complicated MS2/MS3 datasets with database search algorithms to identify unknown proteins (Fellers *et al.* 2015; Cai *et al.*, 2016; Kou *et al.* 2016; Sun *et al.* 2016; Wu *et al.* 2020), but these denatured TDP software packages lack the capability to analyze the isotopically unresolved MS1/MS2' that are characteristic of nMS data. Hence, there is a critical need for a universal software package to address this major challenge in nTDP that can process MS1, MS2, MS2', and MS3 datasets with database search capabilities.

**Figure 1.**
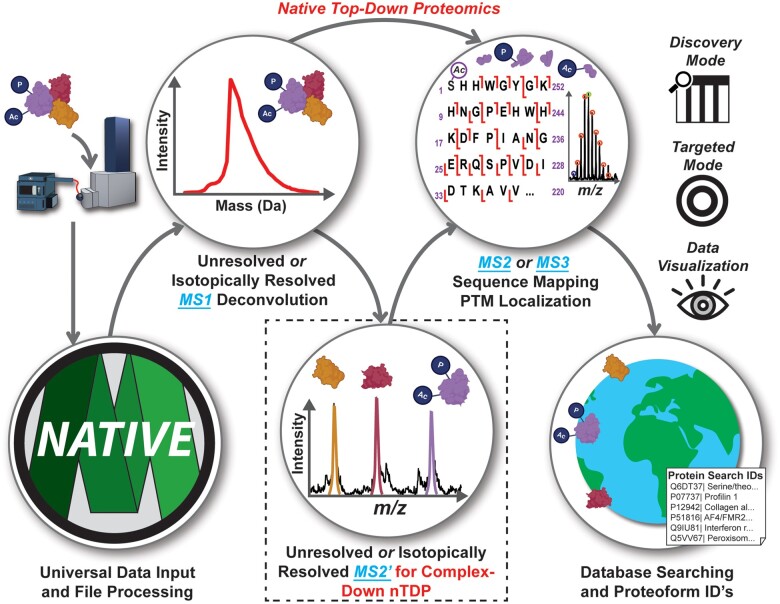
MASH Native provides a universal and comprehensive data processing software for a variety of nTDP analyses. MASH Native is capable of deconvoluting isotopically unresolved protein/protein complex (MS1) and released protein subunits (MS2') spectra, deconvoluting isotopically resolved MS1, MS2', MS2, and MS3 spectra, and performing database searches to identify unknown proteins. MASH Native can process nTDP data in both Discovery Mode and Targeted Mode approaches. It supports various MS file types and integrates multiple deconvolution/search algorithms into one package. MASH Native is a user-friendly software package capable of providing a “one-stop shop” for nTDP data processing.

Herein, we introduce MASH Native, a unified solution for nTDP which can process isotopically unresolved MS1 and MS2' data together with isotopically resolved MS1, MS2, and MS3 deconvolution and database searching (Fig. 1). MASH Native supports various nTDP applications in both targeted mode to characterize known proteins and discovery mode to identify unknown native proteins. It supports various MS file types with different vendor formats and integrates multiple deconvolution/search algorithms into one package. We detail the functions and features of MASH Native and provide examples of processing nTDP data to showcase its capabilities as a “one-stop shop” for nTDP.

## 2 Methods

The MASH Native user interface is a multithreaded Windows desktop application written under a .NET framework environment in Visual Studio using the C# programming language (Wu *et al.* 2020). MASH Native provides universal MS file support through ProteoWizard’s file conversion engine, MSConvert (Chambers *et al.* 2012), and directly imports both vendor-specific MS file types (Thermo *.RAW, Bruker *.d/*.baf/*.ascii) and general file formats (*.mgf, *.mzML, *.mzXML). It is recommended that users perform MASH Native data processing on a computer with at least 4 GB of memory to ensure optimal function of all included algorithms and workflows; however, some deconvolution algorithms may require additional memory for multi-scan, isotopically resolved deconvolution events. The latest version of MASH Native is freely available for download on the MASH website (https://labs.wisc.edu/gelab/MASH_Explorer/MASHNativeSoftware.php) along with licensing information, and written and video user tutorials (also included in the “Supporting Documents for Users” section of the Supplementary Information). All data files used to generate these tutorials are freely available for download on MassIVE as a complete submission (MSV000091693, doi: 10.25345/C5NP1WV0N).

## 3 Results

MASH Native software can deconvolute both isotopically resolved and isotopically unresolved data at the MS1, MS2, and MS3 level and enables database searching of nTDP results (Fig. 1 and Supplementary Table S1). It can process nTDP, nMS, and complex-down proteomics data using multiple deconvolution and database search algorithms with flexible data output options (Supplementary Fig. S1). It also maintains the functions and capabilities previously developed for denaturing TDP so users can process both nTDP and TDP in the same software. To address challenges with low signal-to-noise (S/N) ratios of intact and fragment mass spectra, MASH Native includes a variety of spectral summing algorithms that may be applied prior to data processing workflows (Supplementary Figs S2 and S3). To deconvolute isotopically unresolved MS1 spectra, MASH Native includes UniDec (Marty *et al.* 2015), a powerful deconvolution algorithm, to characterize both isotopically unresolved and isotopically resolved nMS data (Supplementary Fig. S4). Isotopically resolved spectral deconvolution can also be performed in MASH Native (Supplementary Fig. S5), including TopFD (Kou *et al.* 2016), MsDeconv (Liu *et al.* 2010), eTHRASH (Horn *et al.* 2000), and pParseTD (Yuan *et al.* 2012). Users may also import previously deconvoluted results from external deconvolution algorithms, such as FLASHDeconv (Jeong *et al.* 2020), ProMEX (Park *et al.* 2017), or Maximum Entropy (Ferrige *et al.* 1991). Deconvolution results of separate deconvolution workflows can be combined into a single output table, allowing users to view MS1, MS2, and MS3 results simultaneously and combine multiple deconvolution types to improve protein sequence coverage (Mcilwain *et al.* 2020). Results of deconvolution may be searched against a user-selected *.FASTA file or user-defined protein sequence with TopPIC (Kou *et al.* 2016), MS-Align+ (Liu *et al.* 2012), or pTop (Sun *et al.* 2016) to identify proteoforms in a complex mixture. Search results are reported as both gene-level and proteoform-level identifications. Identified proteoforms are scored and ranked, with scoring techniques varying for each algorithm (Liu *et al.* 2012; Kou *et al.* 2016; Sun *et al.* 2016; Basharat *et al.* 2020). Search results generated through MASH Native or from additional search tools such as MSPath-FinderT (Park *et al.* 2017) may then be imported in MASH Native to view identifications, generate fragment ion maps, view fragment ions, and validate for all identified proteins and proteoforms.

### 3.1 Discovery mode workflows

To facilitate high-throughput data analysis, user-defined MASH Native processing workflows can be designed, saved, and queued to allow batch processing of data files using two different approaches: Discovery and Targeted Mode. Discovery Mode facilitates identification of unknown proteins though database searching, a critical processing feature absent from current nMS or native top-down software tools. This mode combines MS1 processing with isotopically resolved MS2 or MS3 deconvolution and database searching in a single workflow for nTDP datasets (Supplementary Fig. S6). To demonstrate MASH Native Discovery Mode for data processing, we accessed and reanalyzed data files from a previously published nTDP dataset of endogenous protein complex previously published by Kelleher and co-workers (MassIVE dataset # MSV000080328) (Skinner *et al.* 2018a). The workflow to identify and characterize subunits of this complex is shown in Supplementary Fig. S6A. Deconvolution of both the MS1 and MS2' spectra by UniDec finds the intact complex mass and released subunit masses. Subsequent isotopically resolved MS3 deconvolution by eTHRASH and database searching with TopPIC identified the two subunits and localized modifications sites on each subunit. This underlines that MASH Native is capable of analyzing complex nTDP data in the Discovery Mode. To identify novel complexes using a complex-down approach, users must begin at the MS3 level by database searching. Next, identified subunits are matched to associated MS2' spectra with intact subunit masses to protein complex interactors. Finally, users must match the detected MS1 mass by testing different stoichiometries of each detected subunit to determine complex stoichiometry and composition. Automation of this process will eliminate the need for manual testing of novel complexes in future MASH Native releases.

### 3.2 Targeted mode workflows

Targeted mode allows users to comprehensively analyze native top-down or complex-down data for a known protein/protein complex, confirm results generated in Discovery Mode, or potentially find new possible complex associations with database searching. At the MS1 and MS2' level, MASH Native enables isotopically unresolved and isotopically resolved native deconvolution through UniDec (Marty *et al.* 2015). Deconvolution and searching of MS2 or MS3 data in targeted mode may be performed using all high-resolution deconvolution algorithms and database search options (vide supra). We have used MASH Native to process a native top-down MS dataset of the bovine glutamate dehydrogenase hexamer previously published by Loo and coworkers (Li *et al.* 2018) to demonstrate the utility of this targeted workflow (Supplementary Fig. S7). MASH Native allowed isotopically unresolved MS1 deconvolution and isotopically resolved MS2 deconvolution along with sequence mapping and data visualization in a single software package (Supplementary Fig. S7). Recently, our group has demonstrated the utility of MASH for targeted analysis in a complex-down workflow for a native cysteine-linked antibody-drug conjugate (ADC) (Supplementary Fig. S8) (Larson *et al.* 2021). The presence of intrachain disulfide bonds limits the fragmentation efficiency of the ADC and reduces sequence coverage by terminal fragment assignment. MASH Native incorporates searching and assignment of internal fragment ions, increasing sequence coverage and revealing sequence coverage of regions bounded by disulfide bonds (Supplementary Fig. S9) to provide additional higher-order structural information for proteins and complexes (Lantz *et al.* 2021, 2022).

## Conclusion

MASH Native provides a unified software solution for the analysis of a variety of complex nTDP data for the first time. As a freely available and universal processing tool, MASH Native is a “one-stop shop” for nTDP data processing that can handle a variety of complex nTDP datasets including isotopically unresolved and isotopically MS1, MS2', MS2, and MS3 in both Discovery and Targeted Modes with database search algorithms as well as data visualization and validation in a user-friendly interface. It can process raw data from various vendor formats and integrates multiple deconvolution/search algorithms into one package. MASH Native has been well-recognized since its release on April 7, 2022 (Liu *et al.* 2022), and downloaded more than 1400 times by users all around the world (66% from North America, 22% from Europe, 7% from Asia, 4% from Oceania, 0.6% from South America, and 0.4% from Africa) (Supplementary Fig. S10). As the nTDP community gains momentum to grow rapidly, MASH Native will play an increasingly important role to streamline nTDP data processing and accelerate the use of nTDP in structural biology and biomedical applications.

## Supplementary Material

btad359_Supplementary_Data
